# Fluorescence of Alexa Fluor Dye Tracks Protein Folding

**DOI:** 10.1371/journal.pone.0046838

**Published:** 2012-10-08

**Authors:** Simon Lindhoud, Adrie H. Westphal, Antonie J. W. G. Visser, Jan Willem Borst, Carlo P. M. van Mierlo

**Affiliations:** 1 Laboratory of Biochemistry, Wageningen University, Wageningen, The Netherlands; 2 Microspectroscopy Centre, Wageningen University, Wageningen, The Netherlands; University of South Florida College of Medicine, United States of America

## Abstract

Fluorescence spectroscopy is an important tool for the characterization of protein folding. Often, a protein is labeled with appropriate fluorescent donor and acceptor probes and folding-induced changes in Förster Resonance Energy Transfer (FRET) are monitored. However, conformational changes of the protein potentially affect fluorescence properties of both probes, thereby profoundly complicating interpretation of FRET data. In this study, we assess the effects protein folding has on fluorescence properties of Alexa Fluor 488 (A488), which is commonly used as FRET donor. Here, A488 is covalently attached to Cys69 of apoflavodoxin from *Azotobacter vinelandii*. Although coupling of A488 slightly destabilizes apoflavodoxin, the three-state folding of this protein, which involves a molten globule intermediate, is unaffected. Upon folding of apoflavodoxin, fluorescence emission intensity of A488 changes significantly. To illuminate the molecular sources of this alteration, we applied steady state and time-resolved fluorescence techniques. The results obtained show that tryptophans cause folding-induced changes in quenching of Alexa dye. Compared to unfolded protein, static quenching of A488 is increased in the molten globule. Upon populating the native state both static and dynamic quenching of A488 decrease considerably. We show that fluorescence quenching of Alexa Fluor dyes is a sensitive reporter of conformational changes during protein folding.

## Introduction

The manner by which proteins attain their functional conformation has been a major puzzle in Biochemistry since the seminal experiments of Anfinsen [1]. Combinations of theory, simulation and experiment have led to the concept of funnel-shaped folding energy landscapes [2], [3], [4]. In this concept, unfolded protein molecules descend along a funnel describing the free energy of folding, until the folding molecules reach the state that has the lowest free energy, which is the native state. Presence of kinetic traps and barriers in a folding energy landscape can lead to population of partially folded or misfolded states in the ensemble of folding molecules. The corresponding folding intermediates may form *en route* to the native state (i.e., they are on-pathway), or may require significant unfolding before the native state can be reached (i.e., they are off-pathway). Often, these intermediates are molten globules. Molten globules are ensembles of interconverting conformers with significant amounts of secondary structure, but lack the tertiary packing characteristics of native proteins [5], [6]. Formation of these aggregation-prone molten globules is linked to the development of various devastating pathologies [7], [8].

Various approaches exist to experimentally tackle protein folding [9]. Frequently, fluorescence spectroscopy is chosen, because this technique is versatile and very sensitive. Several fluorescence read-outs can be used to track protein folding [10], [11]. For instance, fluorescence intensity of tryptophan residues reports on folding-induced changes in the polarity of the microenvironment of these residues. Emission spectra can shift to shorter or longer wavelengths upon folding, and fluorescence anisotropy is folding-state dependent [12], [13]. Upon appropriate labeling of proteins with bright fluorescent dyes, such as Alexa fluorophores [14], even folding of single-molecules can be detected [15], [16], [17], [18].

Through recording of changes in FRET, folding-induced conformational alterations can be monitored [19], [20]. FRET is the distance dependent transfer of electronic excitation energy from an excited donor fluorophore to an acceptor chromophore through non-radiative dipole-dipole coupling [21], [22]. The efficiency of FRET depends on the inverse 6^th^ power of the distance between donor and acceptor. Introduction of an acceptor fluorophore in the vicinity (i.e., up to ∼10 nm) of a donor fluorophore establishes an additional relaxation path for the excited donor, resulting in a decreased fluorescence lifetime of the donor. Regarding FRET studies, ideally, only changes in inter-dye distance lead to altered fluorescence properties of donor and acceptor. However, apart from FRET, changes in protein conformation potentially also affect fluorescence intensities and lifetimes of the probes involved.

**Figure 1:**
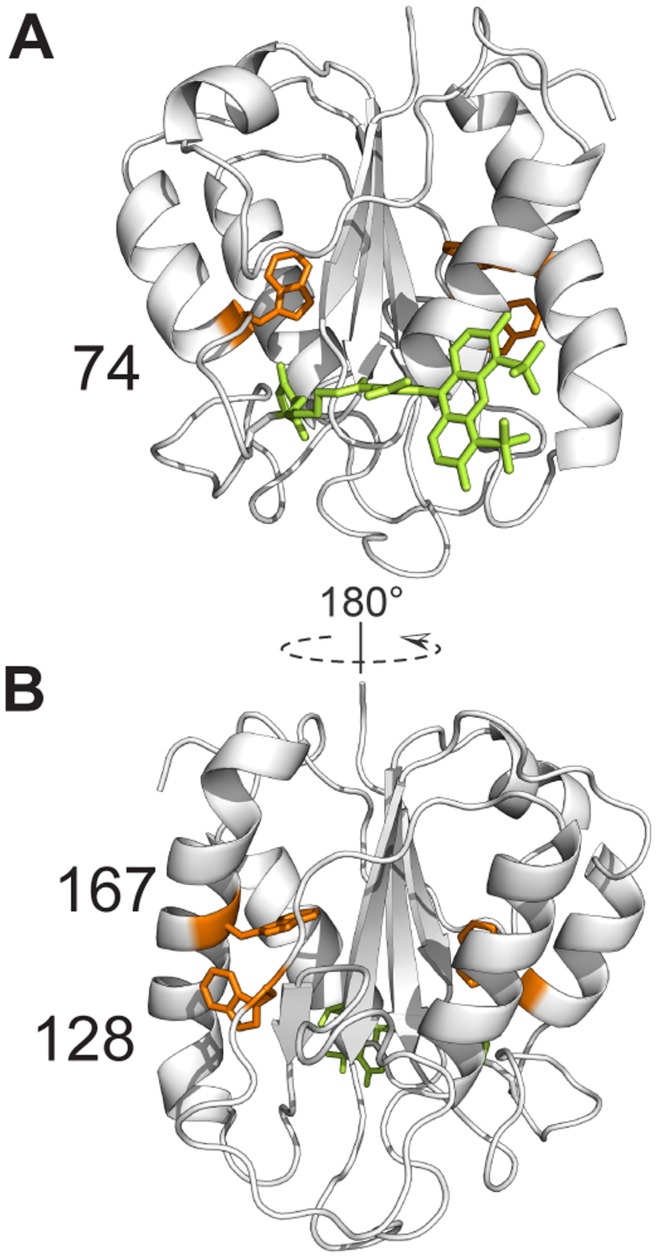
Cartoon model of donor-only labeled apoflavodoxin (i.e., flavodoxin without FMN). A488 is shown in bright green and is attached to residue 69. Flavodoxin’s three tryptophan residues (i.e., Trp74, Trp128 and Trp167) are shown in orange. The model in B is rotated by 180 degrees along the z-axis, compared to the model shown in A. Cartoon models are generated with PyMOL (Schrödinger, LLC, Palo Alto, Ca, USA) using the crystal structure of *A. vinelandii* flavodoxin (pdb ID 1YOB [41]) and the molecular structure of A488, as provided by Invitrogen. Apoflavodoxin strongly resembles flavodoxin, except for dynamic disorder in the flavin-binding region.

Often, Alexa Fluor 488 (A488) is used as donor fluorophore in FRET-detected protein folding (see e.g., [17], [23], [24]), since it is photostable, readily excited at 488 nm, and has a high fluorescence quantum yield. In this study, we assess the effects protein folding has on fluorescence properties of A488 that is covalently attached to Cys69 of apoflavodoxin from *A. vinelandii*. This protein is referred to as A488-apoflavodoxin (Fig. 1). Based on extensive stopped-flow fluorescence spectroscopy data, we showed that apoflavodoxin kinetic folding is described by [25], [26]:
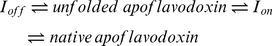
(Scheme1)


Analysis of kinetic folding data shows that on-pathway intermediate *I_on_* is highly unstable [25]. Due to its extremely low population, this folding state is not detected in equilibrium folding experiments (i.e., experiments in which denaturant is added to apoflavodoxin and subsequently the protein is allowed to reach thermodynamic equilibrium before acquisition of spectroscopic data). In contrast, because off-pathway intermediate *I_off_* is stable, this folding state significantly populates in equilibrium folding experiments. Hence, equilibrium folding of apoflavodoxin is described by:
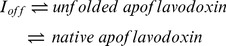
(Scheme2)


Non-covalent binding of flavin mononucleotide (FMN) to native apoflavodoxin is the last step in flavodoxin folding and during global unfolding of flavodoxin release of FMN happens first [27]. Flavodoxin functions in *A. vinelandii* as a one-electron transporter with a strongly negative redox potential [28]. NMR spectroscopy revealed that the off-pathway character of *I_off_* arises from formation of native and non-native helices in unfolded apoflavodoxin and their subsequent non-native docking [29], [30], [31]. *I_off_* is a molten globule, and aggregates severely under conditions that mimic macromolecular crowding inside cells [23].

Here, we show that fluorescence of A488 increases significantly upon folding of A488-apoflavodoxin. This observation potentially affects FRET data of folding-induced conformational changes and their interpretation. To illuminate the molecular sources of the changes in fluorescence quenching, we use steady state and time-resolved fluorescence spectroscopy.

## Materials and Methods

### Engineering, Expression and Purification of Flavodoxin

Wild-type flavodoxin (i.e., flavodoxin containing Cys69 as single cysteine residue) was expressed in *Escherichia coli* TG2 cells, grown in Terrific Broth medium, and was purified according to well-established procedures [32]. To avoid oxidation of cysteine, dithiothreitol (DTT) was present during protein purification.

The buffer used in all experiments with purified protein was 100 mM potassium pyrophosphate, pH 6.0, unless otherwise mentioned.

### Labeling of Cys69 with A488

To optimize accessibility of Cys69 for labeling, flavodoxin was unfolded in 6 M guanidine hydrochloride (GuHCl; Fluka), 100 mM potassium pyrophosphate, pH 7.0. Subsequent addition of 10-fold molar excess of Alexa Fluor 488 C_5_ maleimide (i.e., A488 (Invitrogen)), for a period exceeding 60 minutes, led to labeling of Cys69. The resulting A488 labeled apoflavodoxin molecules (A488-apoflavodoxin) were separated from unreacted label, FMN and GuHCl, using gel filtration with a Superdex75 10/30 HR column (Pharmacia). To determine the concentration of dye-labeled protein stock, absorption spectra were acquired on an HP-8453 diode array spectrophotometer. Dye-labeled protein stock was divided into 50 µL aliquots, frozen in liquid nitrogen, and stored at −80°C.

To assess the quality of A488-apoflavodoxin, we followed fluorescence quenching of FMN. Upon binding to the protein, fluorescence of FMN severely quenches. In addition, we acquired far-UV circular dichroism (CD) spectra of apoflavodoxin and A488-apoflavodoxin.

### Denaturant-dependent Equilibrium Folding

Steady-state fluorescence and circular dichroism (CD) were used to follow denaturant-dependent equilibrium folding of A488-apoflavodoxin. Each data point was acquired at 25°C using 2 µM protein in the appropriate GuHCl concentration. Steady-state fluorescence measurements were done on a Cary Eclipse fluorescence spectrophotometer (Varian). Several combinations of excitation and emission wavelengths were used. Tryptophan fluorescence was measured at 330, 340, 350 and 360 nm, upon excitation at 280 nm. A488 was excited at 475 nm and 493 nm, and fluorescence emission was measured at 515 nm. Fluorescence signals were recorded for 7.125 seconds and averaged. Excitation and emission slits were set to a width of 5 nm. CD signals were acquired by use of a J715 spectropolarimeter (Jasco). Denaturant-dependent protein folding was followed in a 1 mm quartz cuvette by measuring ellipticities at 222, 225 and 255 nm, and the corresponding signal was averaged over 3 min/wavelength with a 1s data interval. The averaged ellipticity at 255 nm was subtracted from the averaged ellipticities measured at 222 and 225 nm.

We also acquired time-resolved fluorescence of a denaturant-dependent equilibrium folding series of 62.5 nM dye-labeled apoflavodoxin. Use was made of the time-correlated single photon counting set-up described elsewhere [33]. Pulse duration was 0.2 ps, pulse energies were at the pJ level and the repetition rate of pulses was 3.8×10^6^ Hz. Decay curves were constructed by collecting photons in 4096 channels of a multi-channel analyzer using a channel time spacing of 5.0 ps. A488 fluorescence lifetimes were measured using excitation at 450 nm, the fluorescence emission was filtered through a 512.2±13.4 nm interference filter with a 3 mm GG 475 cut-off filter (all filters are from Schott, Mainz, Germany). Background fluorescence was measured using the same conditions. The dynamic instrumental response function was determined using a freshly made solution of erythrosine B in water as reference compound (OD_450 nm_ is 0.1; the fluorescence lifetime *τ* is 89 ps at 20°C [33]). Fluorescence decay curves were analyzed using the TRFA data processor (SSTCenter, Minsk, Belarus).

To avoid protein adsorbing to surfaces, Tween-20 was added to all solutions to a final concentration of 0.0035% (w/v). This addition does not affect apoflavodoxin stability, since no change in thermal midpoint of apoflavodoxin unfolding is observed. Prior to measurements, samples stood for 16 to 24 hours in the dark at 25°C, and were at equilibrium. Refractometry was used to determine the GuHCl concentration in each individual sample [34].

### Thermodynamic Analysis of Equilibrium Folding Data

A three-state model (equations 1 to 5), was globally fitted to equilibrium folding data of A488-apoflavodoxin, monitored by fluorescence emission of tryptophan and of A488, and by far-UV CD, using ProFit (QuantumSoft, Zürich, Switzerland):
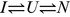
(1)

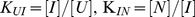
(2)

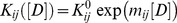
(3)

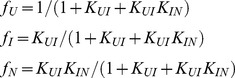
(4)

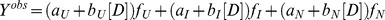
(5)in which *U*, *I* and *N* represent unfolded protein, off-pathway folding intermediate and native apoflavodoxin, respectively, *K_ij_* is the equilibrium constant of the i-j equilibrium, *m_ij_* is the constant that describes the denaturant concentration-dependence of *K_ij_*, superscript *0* designates the parameter at zero denaturant concentration, [D] is the denaturant concentration, *f_i_* is the fractional population of state *i* at a particular denaturant concentration, *Y^obs^* is the observed spectroscopic signal, *a_i_* is the spectroscopic property of state *i* at zero denaturant concentration, and *b_i_* is the constant describing the denaturant concentration-dependence of the spectroscopic signal of state *i*.

**Figure 2:**
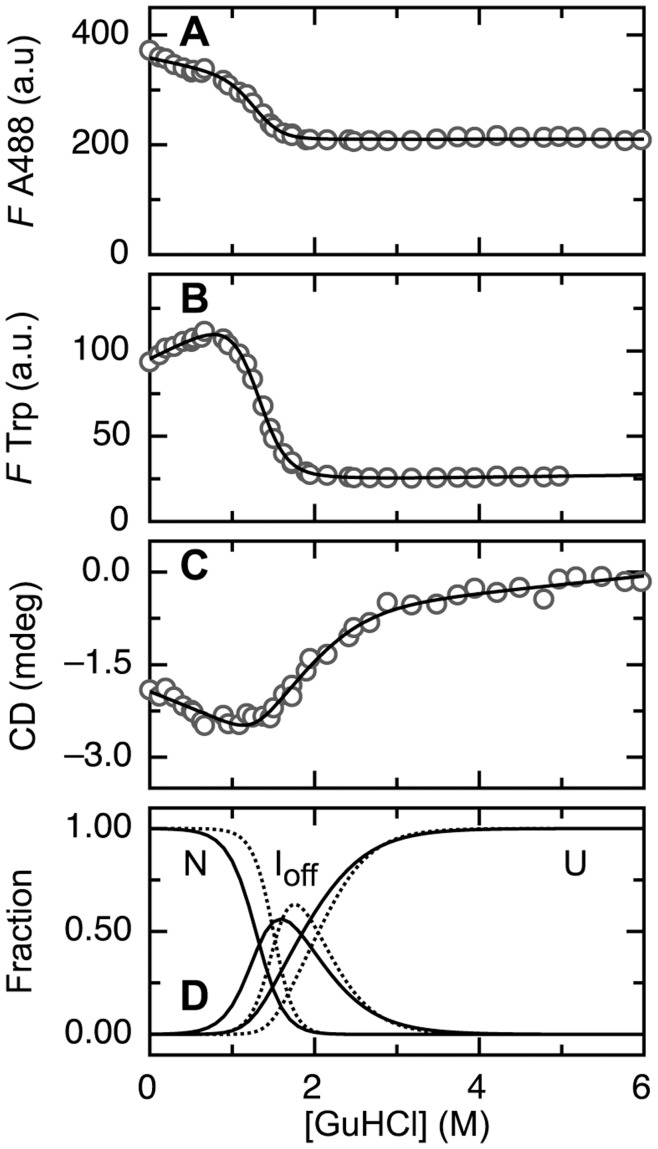
An intermediate populates during denaturant-dependent equilibrium folding of A488-apoflavodoxin. (A) Fluorescence emission intensity of A488 at 515 nm upon excitation at 475 nm. (B) Fluorescence emission intensity of tryptophan at 330 nm upon excitation at 280 nm. (C) CD at 222 nm. Solid lines in panels A to C are the result of a global fit of a three-state model of equilibrium folding to all data acquired (see Materials and Methods and Results sections). (D) Denaturant-dependence of the populations of native (N), off-pathway intermediate (*I_off_*) and unfolded (U) A488-apoflavodoxin (solid lines) and of apoflavodoxin (dotted lines), respectively. Protein concentration is 2 µM in 100 mM potassium pyrophosphate, pH 6.0, and data are recorded at 25°C.

The denaturant-dependence of the spectroscopic parameters of the off-pathway folding intermediate (*b_I_* in equation 5 cannot be accurately determined because the corresponding folding state populates only in a small range of GuHCl concentrations. Therefore *b_I_* is set to zero in the global fit procedure [25]. Each individual data point was weighted by the square of the corresponding standard error during the global fit procedure.

## Results and Discussion

### Alexa Fluor 488 Fluorescence is a Reporter of Apoflavodoxin Folding

To optimize accessibility of Cys69, we unfold flavodoxin in 6 M guanidine hydrochloride. Subsequent addition of A488 leads to labeling of this amino acid residue. Upon removal of denaturant, unfolded A488-apoflavodoxin autonomously folds to native dye-labeled apoprotein, because apoflavodoxin unfolding is reversible [32]. Subsequent addition of FMN leads to full reconstitution of dye-labeled holoprotein and severe quenching of FMN fluorescence intensity (data not shown). Hence, coupling of A488 to Cys69, which resides in the flavin-binding region of the protein, does not impede the ability of apoflavodoxin to bind the FMN cofactor. Far-UV CD spectra of A488-apoflavodoxin and apoflavodoxin are similar (data not shown), which further substantiates that the conformational properties of A488-apoflavodoxin and apoflavodoxin are alike.

**Table 1:** Thermodynamic parameters extracted from a global fit of a three-state model to folding data of apoflavodoxin [25] and of A488-apoflavodoxin (see Fig. 2 and Results section).

	Apoflavodoxin	A488-apoflavodoxin		Apoflavodoxin	A488-apoflavodoxin
Δ*G_UI_* (kcal/mol)	3.74±0.49	2.67±0.62	*m_UI_* (kcal mol^−1^ M^−1^)	−1.83±0.19	−1.45±0.25
Δ*G_IN_* (kcal/mol)	6.70±0.17	4.27±0.13	*m_IN_* (kcal mol^−1^ M^−1^)	−4.40±0.11	−3.21±0.12
Δ*G_UN_* (kcal/mol)	10.45±0.52	6.94±0.63	*m_UN_* (kcal mol^−1^ M^−1^)	−6.23±0.23	−4.66±0.28

Δ*G_ij_* is the difference in free energy between species *i* and *j* at 0 M denaturant, and *m_ij_* is the dependence of Δ*G_ij_* on denaturant concentration. Errors shown are standard errors.

We determined denaturant-dependent folding curves of 2 µM A488-apoflavodoxin by acquiring (i) fluorescence emission of A488 at 515 nm (upon excitation at 475 (Fig. 2A) and 493 nm, respectively), (ii) tryptophan fluorescence at 330 (Fig. 2B), 340, 350 and 360 nm (upon excitation at 280 nm), and (iii) CD at 222 (Fig. 2C) and 225 nm. Fluorescence emission of A488 tracks folding of A488-apoflavodoxin (Fig. 2A), because quenching of this fluorescence changes significantly upon going from unfolded A488-apoflavodoxin in 6 M GuHCl to native dye-labeled protein at 0 M denaturant. The folding curve obtained by CD (Fig. 2C) has a transition midpoint that lies at higher concentration of denaturant than the midpoints of the folding curves obtained by fluorescence (Figs. 2A, B) (i.e., 1.84±0.53 and 1.33±0.06 M GuHCl, respectively). This observation implies involvement of a stable intermediate during folding of A488-apoflavodoxin, just as happens for apoflavodoxin folding.

**Figure 3:**
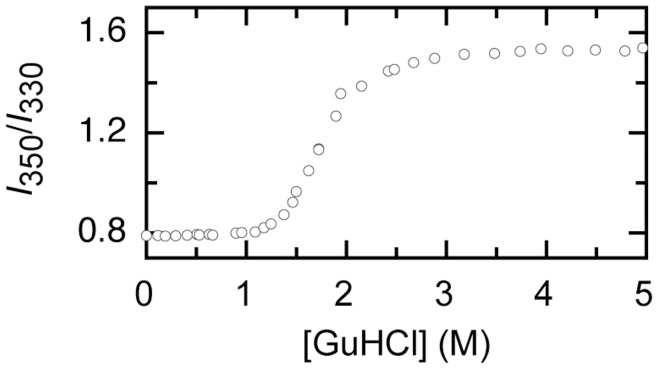
Upon GuHCl-dependent unfolding of A488-apoflavodoxin the ratio of tryptophan fluorescence emission at 350 nm to the corresponding value at 330 nm (i.e., *I_350_*/*I_330_*) alters.

The three-state model for thermodynamic analysis of apoflavodoxin folding (equations 1–5) fits the above-mentioned folding data (Fig. 2). The corresponding thermodynamic parameters (Table 1) show that coupling of A488 to Cys69 predominantly destabilizes native apoflavodoxin. Analogous to modifying cysteine by this attachment, the stability of native apoflavodoxin decreases upon mutating amino acid residues [13], [30], [31], [32]. Yet, just as observed here for A488-apoflavodoxin, folding occurs according to a three-state model, because this is a typical feature of proteins with a flavodoxin-like fold [35].

### The Folding Intermediate of A488-apoflavodoxin is a Molten Globule

Tryptophan fluorescence acquired at 330 nm of A488-folding intermediate and of unfolded A488-apoflavodoxin, both in absence of denaturant, are calculated to be 30% and 24% of the tryptophan fluorescence value that characterizes native A488-labeled protein, respectively. Upon unfolding of apoflavodoxin, λ_max_ of tryptophan fluorescence emission shifts from 329 nm to 352 nm [25]. For A488-apoflavodoxin we measured tryptophan fluorescence at 330, 340, 350 and 360 nm. Due to the expected red shift of λ_max_ upon A488-apoflavodoxin unfolding, the ratio of tryptophan fluorescence emission at 350 nm to the corresponding value at 330 nm (i.e., *I_350_*/*I_330_*) should increase, as Fig. 3 indeed demonstrates. Comparison of Figs. 2D and 3 shows that at the denaturant concentration where *I_off_* populates maximally, λ_max_ must be higher than the value that characterizes native apoflavodoxin. At 1.6 M GuHCl the ratio *I_350_*/*I_330_* has not reached the value that characterizes unfolded protein (Fig. 3). Hence, the intermediate lacks the tertiary side-chain packing characteristics of native A488-apoflavodoxin. Thus, tryptophans of the folding intermediate likely experience a less hydrophobic environment than in native protein. At about 1.8 M GuHCl virtually no native apoflavodoxin molecules are present as judged from fluorescence data (Fig. 2B), but still a significant CD signal is observed (Fig. 2C). Thus, the folding intermediate has a substantial amount of secondary structure, but lacks the tertiary side-chain packing of natively folded apoflavodoxin. This observation is typical for a molten globule-like intermediate. Most likely, just as for apoflavodoxin, the intermediate observed during folding of A488-apoflavodoxin is a molten globule.

**Figure 4:**
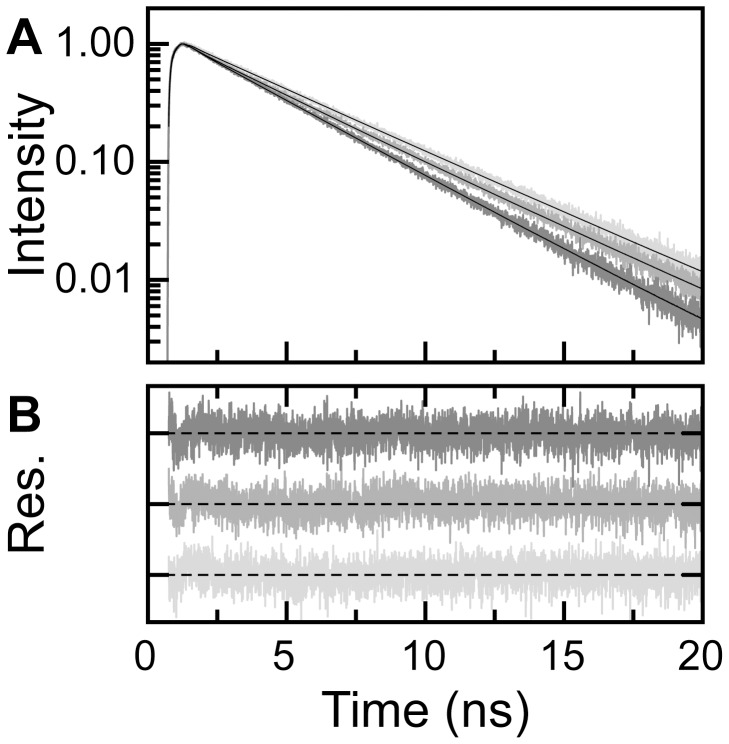
Examples of fluorescence intensity decay curves of A488-apoflavodoxin. (A) Normalized fluorescence decay of A488 of dye-labeled apoflavodoxin at 0.1, 1.5 and 6.9 M denaturant (light grey to dark grey, respectively). Solid lines show the results of a tri-exponential fit of equation 8 to the data. (B) Weighted residuals of the fits.

### Identification of the Sources that Cause Folding-induced Changes in Quenching of A488 Fluorescence

Fluorescence of A488 alters significantly upon folding of A488-apoflavodoxin (Fig. 2A). This phenomenon is due to changes in dynamic and/or static quenching of A488 fluorescence. Static quenching is the result of a non-fluorescent ground-state complex between a fluorophore and quencher that pertains during the excited state lifetime of the fluorophore. When this complex absorbs light it immediately returns to the ground state without emission of a photon. Fluorescence quantum yield *Q_D_* of fluorophores that do not form a complex with quencher is unperturbed. In contrast, dynamic quenching results from transient collisional encounters between a fluorophore and quencher during the lifetime of the excited state, and is a time-dependent process [36]. Thus, dynamic quenching introduces an additional non-radiative decay path from the excited state of A488, and as a result, *Q_D_* as well as the Förster distance *R_0_* decrease. Hence, to be able to quantitatively analyze inter-dye distances in FRET studies of protein folding requires identification of the mechanisms that cause quenching of donor dye, as this study reports for A488. In FRET studies of protein folding one should therefore determine for each folding species whether static and/or dynamic quenching affects fluorescence intensity of donor, and, if necessary, adjust *R_0_*. In the following, we describe how to experimentally identify the sources that cause folding-induced changes in quenching of A488 fluorescence emission of A488-apoflavodoxin.

**Figure 5:**
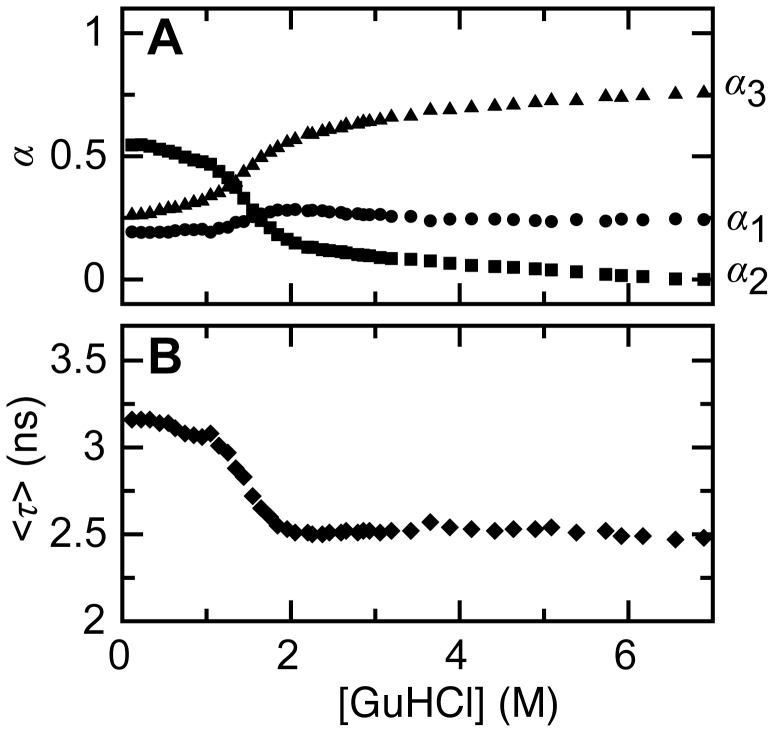
Denaturant-dependencies of normalized amplitudes and average lifetime of the tri-exponential fluorescence decay curves of A488 of dye-labeled apoflavodoxin. (A) Denaturant-dependence of normalized amplitudes *α_1_* (red diamonds), *α_2_* (blue squares) and *α_3_* (orange dots). Corresponding fluorescence lifetimes are: *τ_1_* = 0.439 ns, *τ_2_* = 4.150 ns, and *τ_3_* = 3.161 ns. (B) Denaturant-dependence of average lifetime <*τ>*. To calculate *<τ>,* equation 9 is used.

Dynamic quenching causes a proportional decrease of fluorescence lifetime and intensity of the fluorophore involved. The Stern-Volmer equation describes collisional quenching of fluorescence:
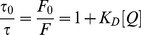
(6)in which *τ_0_* and *τ* are fluorescence lifetimes and *F_0_* and *F* are the fluorescence intensities in the absence and presence of quencher, respectively, *K_D_* is the Stern-Volmer quenching constant (*K_D_  =  k_q_τ_0_*, in which *k_q_* is the bimolecular quenching constant [36]), and [*Q*] is the concentration of quencher.

In contrast, when static quenching is the source of diminished fluorescence intensity, no decrease in fluorescence lifetime is detected [36], because only fluorescent molecules are observed. Therefore, in case of static quenching:
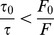
(7)


Thus, by comparing the ratio of fluorescence intensities in the absence and the presence of quencher, versus the ratio of their corresponding fluorescence lifetimes, the sources for quenching of a fluorophore can be identified [37].

With respect to elucidating the contributions of static and dynamic quenching in folding-induced changes of A488 fluorescence, we measured A488 fluorescence intensity decay *I*(*t*) of dye-labeled apoflavodoxin at various denaturant concentrations. By using a sum of discrete exponentials with lifetimes *τ_i_* and amplitudes *α_i_*, a global fit to the data is made according to:
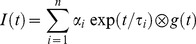
(8)with *g*(*t*) the instrumental response function used for deconvolution of the measured signal. Figure 4 shows examples of A488 fluorescence intensity decay curves obtained for A488-apoflavodoxin. Across the whole denaturant range used, three fluorescence lifetimes (i.e., *τ_1_* = 0.349, *τ_2_* = 4.150, and *τ_3_* = 3.161 ns) describe the decay of A488 fluorescence of dye-labeled apoflavodoxin (global χ^2^ = 1.077). The corresponding amplitudes track the folding of A488-apoflavodoxin (Fig. 5A). Because A488 fluorescence decay is tri-exponential, we need to use the amplitude average fluorescence lifetime [37]:
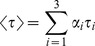
(9)as substitute for fluorescence life times in equation 6. Figure 5B shows that <τ> tracks folding of A488-apoflavodoxin.

**Figure 6:**
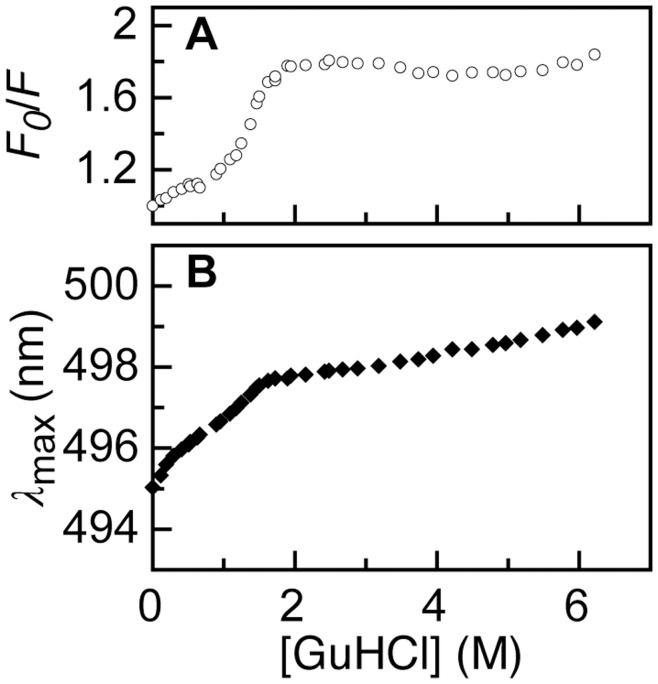
Denaturant-dependencies of *F_0_*/*F* and absorption maximum of A488-apoflavodoxin. (A) Denaturant-dependence of *F_0_*/*F* of A488-apoflavodoxin, using the data of Fig. 2A. (B) The absorption maximum *λ*_max_ of A488-apoflavodoxin shifts from 495 to 499 nm upon going from 0 to 6 M GuHCl.

**Figure 7:**
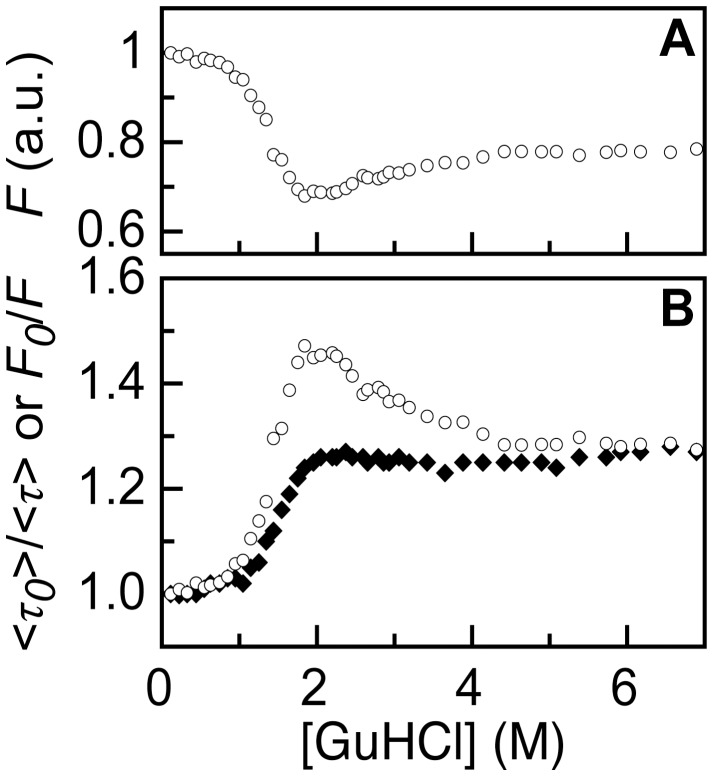
Tracking of changes in static and dynamic quenching of A488 fluorescence upon folding of A488-apoflavodoxin. (A) Denaturant dependence of A488 fluorescence *F*. A488 is excited at its denaturant-dependent fluorescence excitation maximum and A488 fluorescence is recorded at its denaturant-dependent fluorescence emission maximum. (B) Denaturant dependence of *F_0_*/*F* (open dots) and <*τ_0_*>/<*τ>* (filled diamonds), calculated from the data in panel (A) and Fig. 5B, respectively.

We define *F_0_* and <*τ_0_*> as fluorescence intensity and amplitude average fluorescence lifetime of native A488-apoflavodoxin at the lowest denaturant concentration used, respectively. Figure 2A shows that the native baseline of the A488 fluorescence-detected folding curve of apoflavodoxin, which encompasses the GuHCl range of 0 to about 0.7 M, has a rather steep negative slope. As a result, for native protein *F_0_*/*F* changes markedly in this denaturant range (Fig. 6A). In contrast, the amplitude average fluorescence lifetime decreases only slightly in the native baseline (Fig. 5B). To elucidate the origin of the change of *F_0_*/*F* in the native baseline we note that the absorption maximum of A488 shifts from 495 to 499 nm upon going from 0 to 6 M GuHCl (Fig. 6B). This 4 nm shift causes a ∼10% decrease in molar extinction coefficient of A488, and thus in efficiency of excitation of A488 at 475 nm, which is the wavelength used to acquire the data of Fig. 2A. In addition, the fluorescence emission maximum shifts slightly to longer wavelength upon increasing GuHCl concentration.

To avoid both phenomena that cause the change of A488 fluorescence in the native baseline of Fig. 2A and the corresponding increase of *F_0_*/*F*, we determine *F_0_*/*F* by exciting A488 at its fluorescence excitation maximum and record fluorescence at the fluorescence emission maximum of A488 [37]. Figure 7A shows the results of this experiment and Fig. 7B reports the corresponding denaturant dependence of *F_0_*/*F*. Using the fluorescence lifetime data of Fig. 5B, we establish the denaturant dependence of <*τ_0_>/<τ>*, which is shown in Fig. 7B. By comparing the denaturant dependencies of *F_0_*/*F* and <*τ_0_>/<τ>*, the changes in static and dynamic quenching of A488 fluorescence during folding of dye-labeled apoflavodoxin can now be identified.

### Dynamic and Static Quenching of A488 Fluorescence Tracks Protein Folding

Recently, fluorescence emission of Alexa dyes was measured as function of the concentration of the 20 naturally occurring L-amino acids [38]. Tryptophan, tryrosine, methionine, and histidine residues were identified as quenchers of A488. Fluorescence quenching of Alexa 488 originates from photoinduced electron transfer [38], [39] and typically occurs when the distance between fluorophore and quencher is wihin a few Ångstroms. In case of static quenching, quencher and fluorophores are at van der Waals contact distances and photoinduced electron transfer becomes ultrafast [40]. Tryptophan and tyrosine cause similar dynamic quenching of A488. Compared to these amino acids, quenching by methionine and histidine is marginal. In addition, only tryptophan causes considerable static quenching of A488 [38]. Flavodoxin does not contain histidine residues, has one methionine residue (Met30), three tryptophan residues (i.e., Trp74, Trp128 and Trp167), and five tyrosine residues (i.e., Tyr47, Tyr102, Tyr106, Tyr114 and Tyr133). Of these residues, Trp74 most likely causes folding-induced changes in quenching of A488 fluorescence, because this residue is nearest to Cys69 and shielded from solvent in native A488-apoflavodoxin (Fig. 1).

Comparison of the denaturant-dependencies of <*τ_0_>/<τ>* and *F_0_*/*F* reveals that the native baselines of both folding curves have slopes that are equally shallow (Fig. 6B). Thus, addition of denaturant hardly affects A488 fluorescence of native dye-labeled apoflavodoxin. Figure 7B shows that random coil A488-apoflavodoxin, which exists above 6 M GuHCl [29], has similar <*τ_0_>/<τ>*- and *F_0_*/*F*-values, which are both larger than the corresponding values that characterize native protein. Thus, dynamic quenching of A488 in random coil protein is larger than in native apoflavodoxin.

Upon decreasing denaturant concentration from 6 to 2 M GuHCl, *F_0_*/*F* increases considerably, while <*τ_0_>/<τ>* barely alters (Fig. 7B). Apoflavodoxin’s molten globule forms in this denaturant range, whereas the native state of the protein does not populate yet. Consequently, compared to unfolded protein, only static quenching of A488 is enhanced in this folding intermediate. Figure 7B shows that static quenching of A488 exclusively tracks formation of apoflavodoxin’s molten globule. Both static and dynamic quenching of A488 decreases considerably upon increasing the population of the native state by lowering GuHCl concentration below 2 M (Fig. 7B). Clearly, the results of this work show that A488 fluorescence is a sensitive reporter of protein folding.
